# Psychosocial impact of prognostic genetic testing in the care of uveal melanoma patients: protocol of a controlled prospective clinical observational study

**DOI:** 10.1186/s12885-016-2479-7

**Published:** 2016-07-07

**Authors:** Yesim Erim, Jennifer Scheel, Anja Breidenstein, Claudia HD Metz, Dietmar Lohmann, Hans-Christoph Friederich, Sefik Tagay

**Affiliations:** Department of Psychosomatic Medicine and Psychotherapy, Friedrich-Alexander University Erlangen-Nürnberg (FAU), Schwabachanlage 6, 91054 Erlangen, Germany; Clinic for Psychosomatic Medicine and Psychotherapy, LVR Hospital Essen, University of Duisburg-Essen, Virchowstr.174, 45147 Essen, Germany; Department of Ophthalmology, University Hospital Essen, Hufelandstr. 55, 45147 Essen, Germany; Department of Human Genetics, University Hospital Essen, Hufelandstr. 55, 45122 Essen, Germany

**Keywords:** Uveal melanoma, Genetic testing, Psychological distress, Quality of life, Psycho-oncology, Resilience, Social support, Shared decision-making

## Abstract

**Background:**

Uveal melanoma patients with a poor prognosis can be detected through genetic analysis of the tumor, which has a very high sensitivity. A large number of patients with uveal melanoma decide to receive information about their individual risk and therefore routine prognostic genetic testing is being carried out on a growing number of patients. It is obvious that a positive prediction for recidivism in the future will emotionally burden the respective patients, but research on the psychosocial impact of this innovative method is lacking. The aim of the current study is therefore to investigate the psychosocial impact (psychological distress and quality of life) of prognostic genetic testing in patients with uveal melanoma.

**Design and methods:**

This study is a non-randomized controlled prospective clinical observational trial. Subjects are patients with uveal melanoma, in whom genetic testing is possible. Patients who consent to genetic testing are allocated to the intervention group and patients who refuse genetic testing form the observational group. Both groups receive cancer therapy and psycho-oncological intervention when needed. The psychosocial impact of prognostic testing is investigated with the following variables: resilience, social support, fear of tumor progression, depression, general distress, cancer-specific and general health-related quality of life, attitude towards genetic testing, estimation of the perceived risk of metastasis, utilization and satisfaction with psycho-oncological crisis intervention, and sociodemographic data. Data are assessed preoperatively (at initial admission in the clinic) and postoperatively (at discharge from hospital after surgery, 6–12 weeks, 6 and 12 months after initial admission). Genetic test results are communicated 6–12 weeks after initial admission to the clinic.

**Discussion:**

We created optimal conditions for investigation of the psychosocial impact of prognostic genetic testing. This study will provide information on the course of disease and psychosocial outcomes after prognostic genetic testing. We expect that empirical data from our study will give a scientific basis for medico-ethical considerations.

**Electronic supplementary material:**

The online version of this article (doi:10.1186/s12885-016-2479-7) contains supplementary material, which is available to authorized users.

## Background

There are two tumor-biological classes of uveal melanoma, which differ markedly from each other concerning the risk of metastasis [1, 2]. Both tumor classes can be determined either through detection of monosomy 3 in tumor DNA [1], or by a multi-gene expression profile of tumor RNA [2]. Due to the tumor class, the risk of developing metastases varies pronouncedly: In a current study [3], the mortality rates due to metastasis were 13.2 % for tumors with disomy 3 and 75.1 % for tumors with monosomy 3 (median follow-up time of 5.2 years).

Generally, it was found that global quality of life was significantly reduced in patients with malignant uveal melanoma (compared to the healthy norm and other ophthalmological patients, both pre and post treatment), with one half of patients displaying clinically relevant distress pre treatment and one third post treatment [4]. More recent studies concerning quality of life have revealed different improvements and deteriorations after treatment of uveal melanoma: significant decrease of physical functioning and physical role, but on the other hand significant improvement of mental health [5]; furthermore decreases in social functioning, but also in anxiety levels [6].

Prognostic testing of monosomy 3 in uveal melanoma patients aims at determinating the risk of metastasis in patients who have already been diagnosed with cancer. However, (until now) genetic testing does not directly affect treatment decisions [7]. This is in contrast to most of the studies targeting the psychosocial impact of prognostic genetic testing, which are performed with persons at risk for familial cancer (hereditary risk), but not yet diseased. Investigating uveal melanoma patients broadens this field by examining patients who have to decide whether they want to receive very reliable information about their future (prognosis and life perspective), while already being affected with cancer. Due to the dichotomous outcome (disomy 3 = good prognosis, monosomy 3 = poor prognosis), we assume patients to have high levels of involvement and psychological distress. Comparably, a positive test for a predisposition to breast cancer causes psychological reactions similar to those after a manifest diagnosis [8].

In relation to this, additional questions arise concerning patient autonomy and decision making. Concerning the psychological impact of prognostic genetic testing in patients with uveal melanoma, three studies could be identified: In a retrospective study, patients wanted prognostic information even though they were informed that medical care would not be influenced by the result. Furthermore, depressive symptoms and quality of life were found to be independent of a positive test result [9]. Another research group did not find any patients having regrets about prognostic diagnostics (even in the case of poor prognosis) and therefore concluded that there were no harmful effects of the diagnostic testing [10]. In another prospective study [11], patients often did not seem to realize they had to make a decision, for prognostic genetic testing was part of “normal” treatment for them – patients utilized the test because they trusted in what their physicians offered them. The authors concluded that active decision-making and consent procedures are not realistic in acute clinical situations. They even further stated that protecting patients’ interests is the physicians’ responsibility and not delegable to patients.

Moreover, two important issues have to be noted: First, only few respondents strive for an autonomous role in the decision-making process, as often a passive role (particularly in older and less educated patients) is preferred [12]. Second, cancer risks and heredity likelihood were often perceived inaccurately by the counselees. Outcomes like quality of life or current psychological wellbeing could therefore not be predicted by the actually communicated cancer risk, but were mainly mediated or predicted by perceived risk [13]. This might also counteract informed decision-making.

Another important issue is the question of which individuals take the opportunity of genetic testing and/or counseling and which individuals do not (different motivating factors). In a sample of colorectal cancer patients, nonmetastatic cancer, lower perceived risk for cancer recurrence, and greater self-efficacy were associated with greater perceived benefits – whereas perceived barriers were related to cancer-specific psychological distress. Generally, individuals considering the test have positive attitudes towards it and perceive few barriers [14]. Furthermore, the following factors were found to be related to the decision to utilize genetic testing for BRCA 1/2: personal history of cancer, perceiving more benefits than barriers concerning genetic testing, having greater family hardiness, and perceiving a breast cancer diagnosis as associated with fewer negative consequences [15]. Other common motivations for the utilization of prognostic genetic testing are use of more screening offers, reassurance, and taking care of oneself ([16]; breast cancer screening).

## Study aims

The objective of this study is to investigate the psychosocial impact (psychological distress and quality of life) of prognostic genetic testing in patients with uveal melanoma.

This comprises three main issues:

1. Decision-making: Which patients utilize genetic testing? What attitudes predominate in these patients? For this purpose we ask patients about their attitudes towards genetic testing, perceived risk, social support, and sociodemographic data.
2. Psychological distress: How distressing is genetic testing? Should psychological interventions be implemented to reduce pronounced psychological distress? To measure this, we investigate distress and record which patients utilize psycho-oncological interventions.
3. Risk: Which patients are at risk for pronounced psychological distress? To answer this question, we assess resilience, social support, and fear of progression as moderating factors of mental health and stability.

A prospective study is being conducted to investigate (possibly different) trajectories of disease between patients undergoing and not undergoing prognostic genetic testing. In particular, it is possible to examine whether and how patients benefit from knowing the prognosis, even though no medical decisions depend on the test results. These findings should be included in future informed consent. Furthermore, we will be able to overcome shortcomings of previous studies in this field, namely retrospectivity [9], a qualitative approach and small sample sizes [10, 11].

### Hypotheses

1. Sociodemographic and psychological features will moderate the utilization of prognostic genetic testing: Patients with high education, high social support, high resilience, and a previous history of cancer will utilize genetic testing more often than patients with low levels of education, or low social support, or a previous history of cancer.
2. Attitudes towards prognostic testing (attitude scale) will remain stable between the measurement points. Patients with a positive attribution towards genetic testing will give their consent to it more often.
3. Perceived risk of metastasis will change after disclosure of the test result. In approximately 50 % of the patients, individually perceived risk and the objective result of the test will be congruent (congruency means that patients understood the communicated information).
4. Psychological variables will change after prognostic testing and in the further trajectory of the disease:
  - At T1 (initial admission to the clinic, diagnosis), psychological distress levels of patients with uveal melanoma will be significantly increased compared to a norm sample – irrespective of whether they gave their consent to genetic testing (intervention group; IG) or not (observational group; OG).
  - At T2 (day of discharge after surgery), IG-patients and OG-patients will not differ concerning psychological distress, but distress levels will be increased compared to T1 and compared to a norm sample.
  - At T3 (communication of the result, 6–12 weeks after surgery), IG-patients with poor prognosis (IGpp) will have the highest levels of psychological distress and the lowest mental quality of life compared to IG-patients with good prognosis (IGgp) and OG-patients. IPgp-Patients will have the lowest psychological distress and the highest mental quality of life and will be comparable to a norm sample.
  - At T4 (6 months after initial admission), psychological distress in IGpp-patients will be significantly decreased compared to T3, but there will still be significant group differences.
  - At T5 (12 months after initial admission), psychological distress levels in IGpp-patients will be further decreased, but still higher than in IGgp-patients and in a norm sample.
5. Psycho-oncological crisis intervention:
  - Patients with a higher load of psychological symptoms will utilize psycho-oncological interventions more often.
  - Approximately 10 % of the patients will utilize psycho-oncological crisis interventions.
  - Patient satisfaction is expected to be high.

## Methods

### Organizational structure of the study

**Fig. 1:**
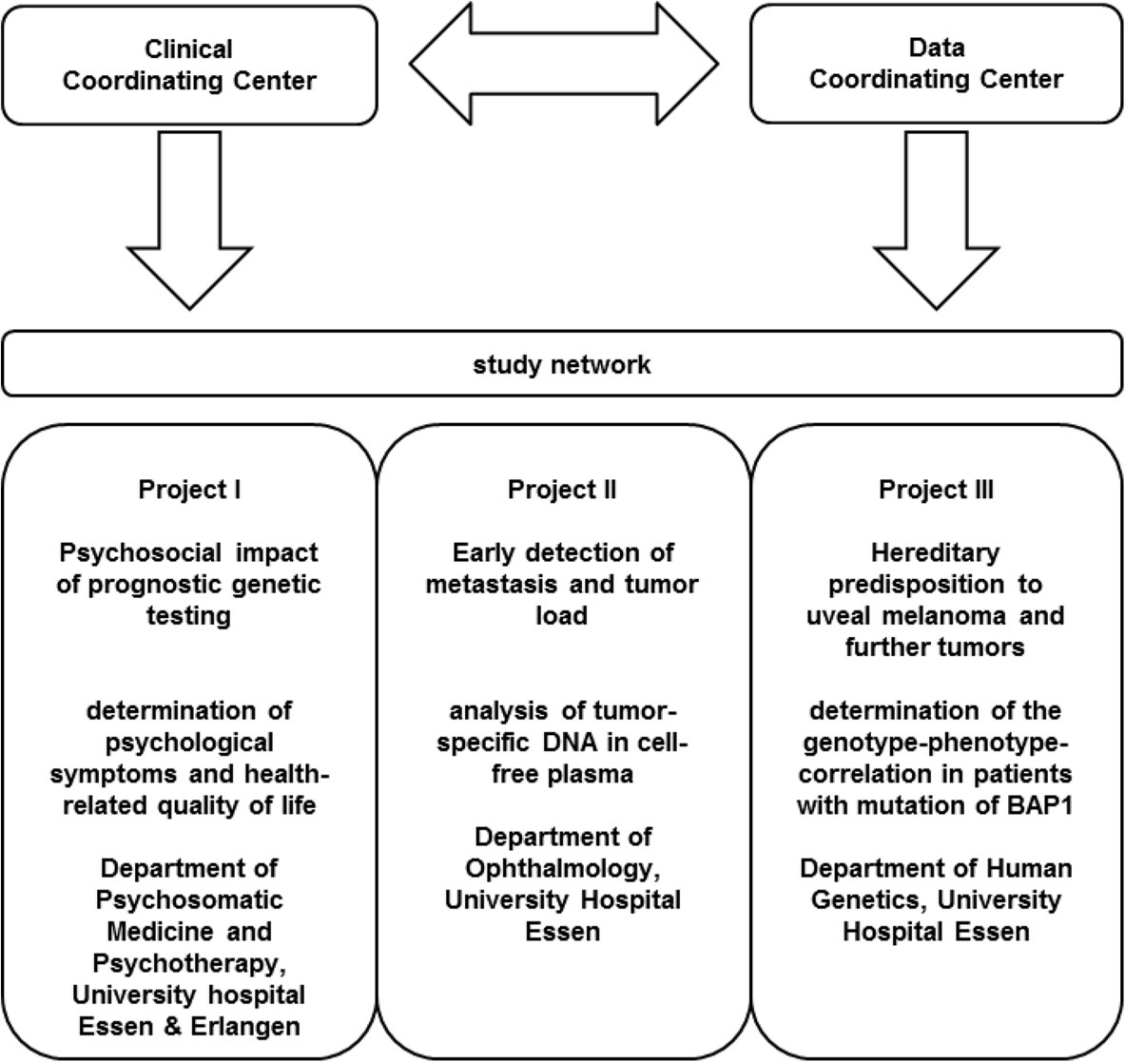
Organizational structure of the study network

This study is conducted within a network of three collaborative investigations (see Fig. 1) funded by the German Cancer Aid. A corporate time table was developed, in which the three projects were embedded (see Table 1).

**Table 1:** Corporate time table of the study network

Assessment	Interval	Project I (psycho-oncology)	Project II (metastasis)	Project III (hereditary predisposition)
T1	Initial admission in the clinic	• Information about the 3 projects and about monosomy 3 diagnostics • medical history (patient and familiy) • psychometric questionnaires		
Surgery (4–6 weeks after initial admission)				
T2	Discharge from hospital after surgery	psychometric questionnaires	plasma sample	
T3	6-12 weeks after surgery (IG); 8 weeks after surgery (OG)	• offer of psycho-oncological support • psychometric questionnaires		Disclosure of test results: • monosomy 3 • tumor disposition
T4	6 months after initial admission	psychometric questionnaires	plasma sample	Follow-up examinations
T5	12 months after initial admission	psychometric questionnaires	plasma sample	Follow-up examinations

### Study population

Patients are included in the study if they are reliably diagnosed with uveal melanoma and if removal of a tumor sample is possible (enucleation or biopsy). Furthermore, they have to be aged between 18 and 90 years, have sufficient knowledge of the German language and give informed consent to take part in the study. Patients are excluded from the study if they have a pre-existing diagnosis of mental disability, psychosis or dementia.

### Recruitment

The sample of this study is a consecutive sample of patients with uveal melanoma undergoing cancer therapy in the Department of Ophthalmology of the University Hospital in Essen, Germany, which is a national center for treatment of patients with ophthalmic tumors. Therefore, more than 170 patients with a first diagnosis of uveal melanoma undergo prognostic genetic testing for monosomy 3 every year.

### Allocation to the study groups

This study is a non-randomized controlled prospective clinical observational trial. Patients are allocated to the groups by their choice (non-randomized) of whether to utilize prognostic genetic testing (intervention group, IG) or not (observationsal group, OG). Blinding is not possible.

### Measurement points

**Fig. 2:**
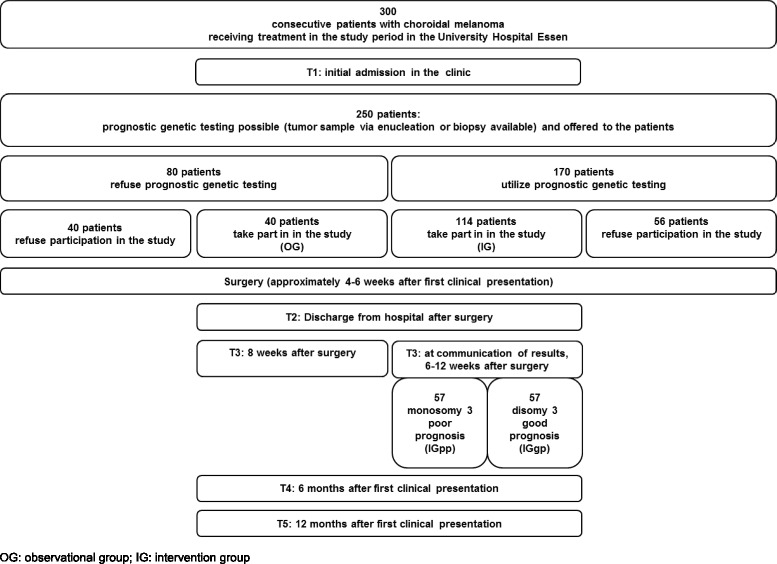
Time flow, expected patient numbers per year

Data are assessed preoperatively (at initial admission in the clinic) and postoperatively (at discharge from hospital after surgery, 6–12 weeks, 6 and 12 months after initial admission); the detailed time flow of the study can be seen in Fig. 2.

### Measures assessed

Exclusively at baseline assessment (initial admission in the clinic): sociodemographic data, medical history and initial protective factors are recorded. For assessment of initial protective factors (resilience and perceived social support), participants complete the short form of the sense of coherence scale [17], the short form of the social support questionnaire [18] and the subscale “social strain” from the long form of the social support questionnaire [19, 20].

The following measures are assessed both at baseline and at all follow-ups: psychological distress, (general and specific) health-related quality of life, attitude towards genetic testing, estimation of the perceived risk of metastasis and utilization of psycho-oncological interventions.

Psychological distress is measured by the Fear of Progression Questionnaire [21, 22], the Hospital Anxiety and Depression Scale (subscale depression, HADS-D, [23]), and the distress thermometer [24]. Specific health-related quality of life is assessed by the EORTC Quality of Life Questionnaire (EORTC QLQ-C30, [25]) and the ophthalmic module of the EORTC QLQ-C30 (EORTC QLQ-OPT 30, [26]). General health-related quality of life is measured by the short version of the Short Form Health Survey SF-36 (SF-12; [27]). Attitudes towards genetic testing are assessed with a modified version of the modified Attitudes Scale [28]. The estimation of perceived risk of metastasis is evaluated with a visual analogue scale (VAS), from 0 to 10. The utilization of psycho-oncological (crisis) interventions (control variable) and patient satisfaction with these interventions is recorded with a documentation form.

Detailed information about several of these measures is provided in the Additional file 1: Appendix. Which measures are assessed at which measurement point can be seen in Table 2.

**Table 2:** Use of different measures at different assessment points

	T1	T2	T3	T4	T5
Resilience	✓	-	-	-	-
Social support	✓	-	-	-	-
Fear of progression	✓	✓	✓	✓	✓
Distress	✓	✓	✓	✓	✓
Depression	✓	✓	✓	✓	✓
Health-related Quality of life	✓	✓	✓	✓	✓
Perceived risk	✓	✓	✓	✓	✓
Attitudes towards prognostic testing	✓	✓	✓	✓	✓
Utilization of psycho-oncological intervention	-	✓	✓	✓	✓

### Psycho-oncological crisis interventions

Patients are informed at T1 that they may utilize psycho-oncological interventions. These crisis interventions follow a resource-oriented approach and are conducted by a clinical psychologist specialized in psycho-oncology. Frequency and kind of crisis intervention, as well as patient satisfaction, are assessed as covariates.

### Statistical analysis plan

Data will be analyzed using the software SPSS for Microsoft Windows®. For descriptive analysis, data will be expressed as mean values, standard deviations, and frequencies; distributional characteristics of all variables will be given. To analyze simple linear relations between variables, Pearson correlations will be calculated. For verification/falsification of our hypotheses (group differences), ANOVAs (post hoc: Scheffé tests), repeated measurement ANOVAs, ANCOVAs, parametric tests (for independent and dependent samples) and non-parametric tests (Chi^2^ tests, Mann–Whitney U-tests, Wilcoxon tests, Kruskal-Wallis tests) will be calculated (according to the respective levels of measurement and sample sizes). For prediction of the outcome variables, multiple regression analyses will be performed. For all tests, a significance level of *p* < 0.05 is predetermined.

### Power considerations and calculation of patient attendance

Calculation of the sample size (GPower) for two-tailed t-tests with dependent samples, effect size = 0.6, probability of error α = 0.05, and power = 0.8 resulted in a sample size of *n* = 24 per group. For two-tailed t-tests with independent samples, the same calculation resulted in *n* = 45 per group. Due to anticipation of dropouts in the study progress, we enlarged this sample size by one third, so the final calculation of the sample size is *n* = 60 per group (*N* = 120). For the calculation of patient attendance see Fig. 2.

### Progress

**Fig. 3:**
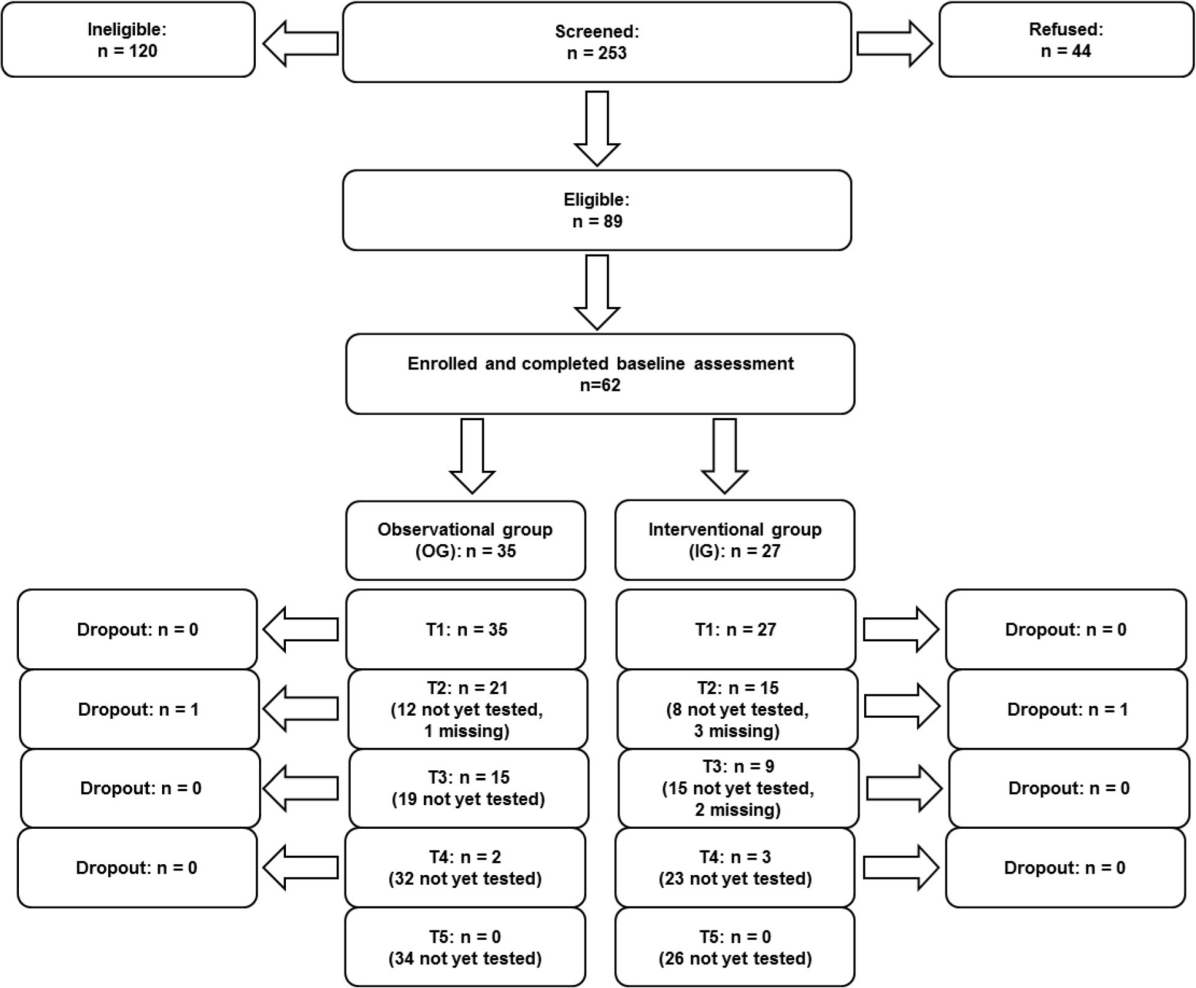
Study flowchart

Patient recruitment started in October 2014. At the moment, 62 patients are already taking part in the study. For detailed information about the number of patients in each group and at each assessment point, please see Fig. 3. Baseline sociodemographic and disease-related data of all IG- and OG-patients included are presented in Table 3.

**Table 3:** Baseline sociodemographic and disease-related characteristics of patients to date

		Oberservational group (OG)	Intervention group (IG)
	n	35	27
Sociodemographic characteristics	Age in years	mean ± sd: 60.03 ± 12.41	mean ± sd: 62.96 ± 12.30
		range: 37–78	range: 40–82
	Sex	female: 16	female: 11
		male: 19	male: 16
	Nationality	German: 34	German: 26
		Polish: 1	Dutch: 1
Disease-related characteristics	Treatment	brachytherapy: 32	brachytherapy: 14
		endoresection: 0	endoresection: 4
		endoresection and Gamma-Knife: 1	endoresection and Gamma-Knife: 1
		cyber-Knife: 0	cyber-Knife: 1
		enucleation: 2	enucleation: 7
	Method of tumor sampling	-	pars plana vitrectomy: 15
			endoresection: 5
			enucleation: 7
	Utilization of psycho-oncological support	support offered by study program: 2	support offered by study program: 2
		other support: 2	other support: 1

## Discussion

In the following sections, the psychosocial impact of genetic counseling, decision-making processes and ethical considerations concerning autonomy and communication shall be discussed within the framework of current research.

### Psychosocial impact of genetic counseling

In two meta-analyses, a significant impact of genetic counseling for familial cancer on anxiety (reduction), and on accuracy of perceived risk and knowledge (both improved) was found, suggesting genetic counseling had no adverse psychological effects [29, 30]. Comparably, beneficial effects of multidisciplinary genetic risk counseling for familial colorectal cancer on psychosocial outcome were found: General anxiety, familial cancer-specific distress and general cancer worries were significantly reduced after genetic counseling [31]. Moreover, distress and depression levels were reduced within the first 6 months after counseling and testing [32], and intrusion and avoidance significantly reduced over time [33]. Furthermore, the authors found passive and palliative coping styles, excessive breast self-examination, and overestimation of breast cancer risk to be predictive of increased long-term distress. In contrast, decreased long-term distress could be predicted by promoting reassuring thoughts.

When comparing carriers and non-carriers, carriers were found to be significantly more distressed from testing and to report increased risk perception levels and surveillance (up to four years) [34]. Furthermore, Meiser (2005) [35] differentiated in their review between individuals having never been affected by cancer and individuals affected by cancer. In the former condition, non-carriers psychologically benefitted significantly, whereas carriers experienced no adverse effects. In individuals affected with cancer, the individual former cancer experience seemed to influence the effects of genetic testing.

While waiting for disclosure of the test result, acute anxiety may emerge [36]. Phelps et al. (2013) [37] investigated the effectiveness of a self-help coping intervention in patients awaiting their genetic result. Intrusive thoughts during the waiting period could be reduced significantly in patients with moderate baseline levels of intrusion. Furthermore, distress levels were decreased in patients with low or moderate intrusive worries at baseline. Yet, no intervention effect on the sample as a whole could be demonstrated, suggesting that patients with clinically high levels of psychological distress might need more intensive psychological treatments. The authors conclude that their intervention could provide help while waiting for test results and feeling uncertain, in various oncological patient groups. Moreover, there seem to be no harmful effects on those patients that are likely to not benefit from the intervention.

Our study was designed to investigate if these findings could also account for uveal melanoma patients (who have already been diagnosed with cancer).

### Decision-making

Affected persons have to weigh the pros and cons of genetic testing when making their decision. Pros are, for example, relief from uncertainty, adjustment of important life decisions and life planning, as well as making decisions about intensified prophylaxis and screening programs. Cons are, for example, risks of persistent psychological distress, such as depressive rumination about one’s individual risk of disease.

It is an important ethical issue, as to how decision-making concerning genetic testing can be improved, such as through educative interventions both for patients and for medical staff. Wakefield et al. (2008) [38] developed a decision aid intervention (specifically for informed decision making concerning genetic testing for hereditary non-polyposis colorectal cancer risk) and tested its efficacy in a randomized controlled trial. Participants of the intervention group experienced less decisional conflict and were better informed and therefore had a better chance of making an informed decision with regard to genetic testing. Furthermore, Ilic et al. (2015) [39] (review, prostate cancer) found improvements in knowledge, reduction in decisional conflict and an increase in decisional satisfaction after utilization of a decision aid intervention. Moreover, Dieng et al. (2014) [40] (review, mainly breast cancer studies) found changes in risk perception level and accuracy after educational interventions in prospective observational studies (n = 28), but not in randomized controlled trials (n = 12). We expect our study to shed further light on questions concerning the influence of decision for or against genetic testing.

### Ethical considerations: Autonomy and communication

Another important research question is the appropriate and correct ethical conduct with information. The right of self-determination and respect for the personal autonomy of the patient form the ethical and legal basis for the demand for information and participation in medical decisions. It is important to note, that the right of self-determination includes also the right of not being informed, because negatively appraised health-related information can cause severe psychological distress. So, apart from the ethical principle of autonomy, the principles of non-maleficence and beneficence have to be taken into account for medico-ethical analyses [41]. To minimize psychosocial distress due to prognostic genetic testing, genetic counseling before and directly after communication of the results is recommended by the guidelines of the German Medical Association [42]. This recommendation was taken into account in the elaboration of the German Genetic Diagnostics Act. In a systematic review (93 studies), it was found that most studies about communication of prognosis to cancer patients were conducted in early stages of disease [43]. It remained uncertain which approach of communication would be the best. We expect our study to provide further information about patient autonomy and disclosure of test results.

### Limitations

There are two limitations of our study. First, randomization and blinding is not possible. Second, patients receive different cancer treatments (see Table 3), which might cause differences concerning the psychosocial impact of genetic testing. On the other hand, no differences concerning quality of life, psychological distress, depression and anxiety were found between enucleation and radiotherapy patients or between patients receiving different methods of radiotherapy [44, 45].

### Conclusion

We created optimal conditions for the investigation of the psychosocial impact of prognostic genetic testing and the course of disease, in uveal melanoma patients. The naturalistic design we chose is very suitable for research in the clinical setting, but takes time and effort. We therefore thank the German Cancer Aid for promoting this network of three parallelized collaborative studies from different scientific disciplines, giving the opportunity to investigate uveal melanoma patients from different points of view.

We expect that our findings will be transferable to other oncological entities and clinical syndromes. Furthermore, we expect that empirical data from our study will contribute to the continuing medico-ethical debate on prognostic genetic testing, in the light of patient autonomy and decision-making processes.

## Additional file

Additional file 1: Appendix (DOCX 38 kb)
