# Supplementary material for: Psychosocial impact of prognostic genetic testing in the care of uveal melanoma patients: protocol of a controlled prospective clinical observational study
Source: BMC Cancer. 2016 Jul 7;16:408. doi: 10.1186/s12885-016-2479-7 (PMC4936050; doi:10.1186/s12885-016-2479-7)
Supplement: Additional file 1: — Appendix (DOCX 38 kb) [file 12885_2016_2479_MOESM1_ESM.docx]

**Appendix**

**Initial protective factors: resilience and social support**

For assessment of initial protective factors, participants complete the short-form of the sense of coherence scale (SOC 13, [[1](#_ENREF_1)]), the short form of the social support questionnaire (K-22; [[2](#_ENREF_2)]) and the subscale “social strain” from the long-form of the social support questionnaire (Fragebogen zur sozialen Unterstützung, F-SozU, [[3](#_ENREF_3), [4](#_ENREF_4)]). The SOC-13 [[1](#_ENREF_1)] consists of 13 items and assesses the three subscales comprehensibility, manageability and meaningfulness of the “sense of coherence”. With the K-22 (K-22; [[2](#_ENREF_2)]) perceived social support is assessed. The K-22 consists of 22 items and is composed of three subscales (emotional support, instrumental support, social integration). The F-SozU [[3](#_ENREF_3), [4](#_ENREF_4)] consists of 54 items and is composed of four major scales (emotional support, instrumental support, social integration, social strain) and a global scale. We only use the subscale “social strain” (12 items). Reliability and validity have been tested in different samples and German norm values are available [[5](#_ENREF_5)].

**Psychological distress: Fear of progression, depression, distress thermometer**

Psychological distress is measured by the Fear of Progression Questionnaire [[6](#_ENREF_6), [7](#_ENREF_7)], the Hospital Anxiety and Depression Scale (depression, HADS-D; [[8](#_ENREF_8)]) and the distress thermometer [[9](#_ENREF_9)]. The Fear of Progression Questionnaire [[6](#_ENREF_6), [7](#_ENREF_7)] includes 43 items, which are grouped into five subscales: affective reactions, partner/family, work, loss of autonomy, and coping with fears. The HADS-D [[8](#_ENREF_8)] is an internationally widely used reliable and valid instrument for the assessment of anxiety and depression among medically ill patients [[8](#_ENREF_8), [10](#_ENREF_10)]. Each of the two scales is made up of seven items, which are summed up to a score ranging between 0 to 21. Scores lower than seven are considered clinically not relevant, borderline scores range from eight to 10 points, and clinically relevant anxiety and depression is indicated above 10 points. For our purposes, only the depression scale has been evaluated. The distress thermometer [[9](#_ENREF_9)] is a visual analogue scale from 0 (= no distress) to 10 (= extreme distress) in a thermometer format to rate emotional distress experienced in the last week. With the associated problem list (36 items), problems in five categories are assessed: practical, family, emotional, spiritual/religious, and physical problems.

**Quality of life**

Specific health-related quality of life is measured by the EORTC (European Organisation for Research and Treatment of Cancer) Quality of Life Questionnaire (EORTC QLQ-C30, version 1, [[11](#_ENREF_11)]) and the ophthalmic module of the EORTC QLQ-C30 (EORTC QLQ-OPT 30, [[12](#_ENREF_12)]), general health-related quality of life is measured by the short form of the Short Form Health Survey SF-36 (SF-12; [[13](#_ENREF_13)]; German version). The EORTC QLQ-C 30 [[11](#_ENREF_11)], is a validated, brief, self-reporting, and cancer-specific questionnaire. It consists of 30 items and comprises 14 dimensions of quality of life (functional scales: physical functioning, role functioning, emotional functioning, cognitive functioning, social functioning; symptom scales: fatigue, nausea, vomiting, pain, dyspnea, sleep disturbance, appetite loss, constipation, diarrhea, financial impact) and a global health status / quality of life score. Reliability and validity have been tested and German norm values are available. The EORTC QLQ-OPT 30 [[12](#_ENREF_12)] is designed for patients with uveal melanoma. It consists of 30 items and comprises 8 subscales (ocular irritation, vision impairment, headache, worry about recurrent disease, problems with driving, problems with appearance, functional problems due to vision impairment, problems reading). The SF-12 [[13](#_ENREF_13)] is the short form of the SF-36 Health Survey [[14](#_ENREF_14)], German version [[15](#_ENREF_15), [16](#_ENREF_16)]. It comprises 12 items concerning health-related quality of life (physical and psychological subscales). Reliability, validity, and sensitivity for alterations with time are tested in several studies and norms are available.

**Attitudes towards genetic testing**

Attitudes towards genetic testing are assessed with a modified version of the modified Attitudes Scale [[17](#_ENREF_17)]. [[17](#_ENREF_17)] modified the Attitude Scale [[18](#_ENREF_18)] (attitudes towards genetic testing for breast cancer) for assessing the attitude towards genetic testing for hereditary non-polyposis colorectal cancer (8 pro and 8 con statements on a three-point scale). Two of the pro statements and six of the con statements also apply to patients with uveal melanoma and are used in this study. Two of the con statements were converted in pro statements, so finally, four pro statements (“prefer certainty over uncertainty”; “great relief if I were found not to carry the gene”; “would trust the test”; „not concerned about confidentiality“) and four con statements („prefer not to know my genetic status“; “expect difficulty to cope with a positive test result”; “expect problems/conflicts with the family”; “problems with insurance/employer”) were used.

For OG-patients, the attitudes scale is again modified for assessment points T2-T5. Due to the rejection of genetic testing, some questions about expectations concerning prognostic testing do not make sense. The attitude scale then comprises six items: two items about attitudes concerning rejection of prognostic testing (“regret to have rejected the prognostic testing”, „prefer not to know my genetic status“); two items concerning positive expectations towards prognostic testing (“would trust the test“, „not concerned about confidentiality“); and two items concerning negative expectations concerning prognostic testing (“expect difficulty to cope with a positive test result”, “problems with insurance/employer”).
For IG-patients, the attitudes scale is again modified for assessment points T4 and T5, because patients already know the result of genetic testing at T4 and T5 and some of the items make no sense any more. Eight items are used: two items retrospectively measure attitudes concerning prognostic testing (“great relief if I were found not to carry the gene”; “regret to have consented to prognostic testing”); two items assess negative expectations (“expect difficulty to cope with a positive test result”, “problems with insurance/employer); two items assess positive expectations (“would trust the test“, „not concerned about confidentiality“); two items assess the consequences of prognostic testing (“familial conflicts”, “coping with the result”).

**References Appendix**

1. Antonovsky A. The structure and properties of the sense of coherence scale. Social Science & Medicine. 1993;36:725-33.

2. Fydrich T, Sommer G, Menzel U, Höll B. Fragebogen zur sozialen Unterstützung (Kurzform; SozU-K-22). Zeitschrift für Klinische Psychologie. 1987;16:434-6.

3. Sommer G, Fydrich T. Soziale Unterstützung, Diagnostik, Konzepte, Fragebogen F-SozU. Tübingen; 1989.

4. Sommer G, Fydrich T. Entwicklung und Überprüfung eines Fragebogens zur sozialen Unterstützung. Diagnostica. 1991;37:160-78.

5. Fydrich T, Geyer M, Hessel A, Sommer G, Brähler E. Fragebogen zur Sozialen Unterstützung (F-SozU): Normierung an einer repräsentativen Stichprobe. Diagnostica. 1999;45(4):212-6. doi:10.1026//0012-1924.45.4.212.

6. Herschbach P, Berg P, Dankert A, Duran G, Engst-Hastreiter U, Waadt S et al. Fear of progression in chronic diseases: psychometric properties of the Fear of Progression Questionnaire. J Psychosom Res. 2005;58:505–11.

7. Herschbach P, Dankert A, Duran-Atzinger G, Waadt S, Engst-Hastreiter U, Keller M et al. Diagnostik von Progredienzangst – Entwicklung eines Fragebogens zur Erfassung von Progredienzangst bei Patienten mit Krebserkrankungen, Diabetes mellitus und entzündlich-rheumatischen Erkrankungen in der Rehabilitation [Diagnostic of fear of progression – evaluation of a questionnaire for measurement of fear of progression in patients with cancer, diabetes mellitus and inflammatory rheumatic diseases in rehabilitation] <http://forschung.deutsche-rentenversicherung.de/ForschPortalWeb/rehaDoc.pdf?rehaid=82EF8B8F899B55CEC1256E6A003B729D>. 2013.

8. Herrmann CH, Buss U, Snaith RP. HADS-D: Hospital Anxiety and Depression Scale – German Version. Bern, Germany: Huber; 1995.

9. Roth AJ, Kornblith AB, Batel-Copel L, Peabody E, Scher HI, Holland JC. Rapid screening for psychologic distress in men with prostate carcinoma: a pilot study. Cancer. 1998;82(10):1904-8.

10. Zigmond AS, Snaith RP. The hospital anxiety and depression scale. Acta psychiatrica Scandinavica. 1983;67(6):361-70.

11. Aaronson NK, Ahmedazai S, Bergman B, Bullinger M, Cull A, Duez NJ. The European Organization for Research and Treatment of Cancer QLQ-C30: a quality of life instrument for use in international clinical trials in oncology. J Natl Cancer Inst 1993;85:365-76.

12. Brandberg Y, Damato BE, Kivela T, Kock E, Seregard S. The EORTC ophthalmic oncology quality of life questionnaire module (EORTC QLQ-OPT30). Development and pre-testing (Phase I-III). Eye. 2004;18:283-9.

13. Ware (Jr.) JE, Kosinski M, Keller S. A 12-item shortform health survey: construction of scales and preliminary tests of reliability and validity. Medical Care. 1996;34:220-33.

14. Ware JE, Jr., Sherbourne CD. The MOS 36-item short-form health survey (SF-36). I. Conceptual framework and item selection. Med Care. 1992;30(6):473-83.

15. Bullinger M, Kirchberger I. SF-36. Fragebogen zum Gesundheitszustand. Handanweisung. . Göttingen: Hogrefe; 1998.

16. Bullinger M, Kirchberger I, Ware J. Der deutsche SF-36 Health Survey. Übersetzung und psychometrische Testung eines krankheitsübergreifenden Instruments zur Erfassung der gesundheitsbezogenen Lebensqualität. Zeitschrift für Gesundheitswissenschaften. 1995;3:21-36.

17. Keller M, Jost R, Kadmon M, Wüllenweber H-P, Mastromarino Haunstetter C, Willeke F et al. Acceptance of and Attitude Toward Genetic Testing for Hereditary Nonpolyposis Colorectal Cancer: A Comparison of Participants and Nonparticipants in Genetic Counseling. Dis Colon Rectum 2004;47:153-62.

18. Lerman C, Daly M, Masny A, Balshem A. Attitudes about genetic testing for breast-ovarian cancer susceptibility. Journal of clinical oncology : official journal of the American Society of Clinical Oncology. 1994;12(4):843-50.
